# Acute and Chronic Toxicity of Propylparaben in the Freshwater Snail *Biomphalaria glabrata*: Effects on Survival, Growth, Reproduction, and Histopathology

**DOI:** 10.3390/toxics14030203

**Published:** 2026-02-27

**Authors:** Qingzhi Zhao, Yutong Zhao, Jiyuan Wang, Jialu Xu, Hairun Li, Xinyi Fei, Yijie Zhang, Ruke Wang, Yuqing Shao, Anni Jin, Hao Wu, Lailing Du, Xiaofen Zhang, Huiliang Zou, Hongyu Li, Xiaoling Xu

**Affiliations:** 1 Key Laboratory of Artificial Organs and Computational Medicine in Zhejiang Province, Shulan International Medical College, Zhejiang Shuren University, Hangzhou 310015, China; 2 School of Basic Medicine and Forensic Medicine, Hangzhou Medical College, Hangzhou 310053, China; 3 Huzhou Institute for Food and Drug Control, Huzhou 313000, China

**Keywords:** *Biomphalaria glabrata*, ecotoxicology, histopathology, propylparaben, reproductive toxicity

## Abstract

Propylparaben (PP) is a widely used preservative in cosmetics, pharmaceuticals, and food products, and its potential toxicity to non-target aquatic invertebrates remains a concern. This study used the freshwater snail *Biomphalaria glabrata* as a model organism to evaluate the toxic effects of PP through acute and chronic exposures at embryonic, newly hatched, and adult stages. Acute exposure experiments showed concentration-dependent mortality and developmental inhibition, with LC_50_ values of 36.69 mg/L (embryos, 168 h), 33.48 mg/L (newly hatched snails, 96 h), and 57.05 mg/L (adults, 72 h). Chronic exposure of adult snails to 10–49 mg/L PP for 21 days significantly reduced growth and reproductive output, and no embryo masses were observed at concentrations ≥ 25 mg/L. Histological observations revealed progressive damage to the hepatopancreas and gonads. These results demonstrate that PP induces multiple toxic effects in *B. glabrata*, affecting survival, growth, reproduction, and tissue structure under both acute and chronic exposure conditions. The findings provide experimental evidence for evaluating the ecological risks of paraben contamination in freshwater ecosystems.

## 1. Introduction

Parabens are a group of esters derived from *p*-hydroxybenzoic acid (PHBA), including methylparaben (MP), ethylparaben (EP), propylparaben (PP), isopropylparaben (iPP), butylparaben (BP), and benzylparaben (BeP) [1]. These compounds are typically colorless crystals or white powders with little or no odor or taste [2]. They are readily soluble in alcohol, ether, glycerol, and propylene glycol, but only slightly soluble in water, and their water solubility decreases with increasing alkyl chain length [3]. Meanwhile, parabens possess relatively high lipophilicity and large oil/water partition coefficients [4,5]. Due to their strong antimicrobial activity, parabens have been widely used in pharmaceuticals, food products, and personal care products for over a century [6,7,8]. It has been reported that they are used in more than 22,000 cosmetic formulations, with concentrations up to 0.8% (mixtures) or 0.4% (single compounds) [1]. Approximately 8000 tons of parabens are consumed globally each year [9]. During production, use, and disposal, these compounds inevitably enter the environment and are frequently detected in landfills, wastewater treatment plants, and sewage sludge [10]. In addition, parabens have been found in air, dust, wastewater, surface water, human urine, and tumor tissues [7,8], as well as in paper currency and paper products such as sanitary wipes [11]. Studies have shown that parabens can be efficiently absorbed through the skin; however, intact compounds are rarely detected in blood or urine, suggesting rapid metabolism in the human body [12]. Some paraben metabolites have been suggested to be involved in endocrine disruption in experimental animals, highlighting the need for further studies to clarify their biological effects and potential implications for human health [12].

Understanding and determining the levels of parabens in ecosystems is crucial for assessing their potential ecotoxicity, protecting biodiversity, evaluating human exposure risks, and safeguarding public health [7]. Among parabens, MP and PP are the most widely used in personal care products [13], with PP also being one of the most frequently detected monomers in aquatic ecosystems. Environmental monitoring data have shown that the concentrations of PP vary greatly across different environmental matrices. In influents of wastewater treatment plants, PP levels can be as low as 0.5 ng/L, whereas in certain industrial effluents, they can reach up to 23.593 µg/L [14]. In surface waters, concentrations in rivers typically range from 0.5 [14] to 400 ng/L [15], and approximately 9 ng/L has also been detected in drinking water [16]. In river water and coastal seawater, PP concentrations generally range from several ng/L to several hundred ng/L [9]; PP has also been detected in sediments and sewage sludge at levels of ng/g dry weight. However, due to differences in environmental media, such data are not directly comparable to concentrations measured in water [13]. Notably, PP concentrations are significantly elevated in wastewater systems located near pollution sources. For instance, the mean concentration of PP in hospital wastewater in Greece has been reported to range from 16 to 77 µg/L, with a maximum value of 108 µg/L [17]; similarly, levels up to 73.4 µg/L have been detected in influents of wastewater treatment plants in South Africa [18]. In Spain, PP concentrations in raw wastewater are typically within the range of 0.3–10 µg/L, where PP and MP are the predominant detected parabens [19]. More extreme cases have shown that in certain contaminated waters, the measured concentrations of MP can exceed 0.52–0.56 mg/L, suggesting that under conditions of strong source pressure, some parabens may reach peak levels in the mg/L range [20]. In addition, process and removal efficiency studies often employ 10–50 mg/L of MP, EP, or PP as simulated influent concentrations from cosmetic or pharmaceutical wastewater, reflecting the potential for short-term high-concentration discharges in real scenarios [21].

Currently, no specific regulatory limits have been established for PP concentrations in surface waters or drinking water under major environmental regulatory frameworks, including the European Union Water Framework Directive (2000/60/EC) [22] and the U.S. Environmental Protection Agency’s water quality standards [23]. Although certain regulatory systems provide general frameworks for the assessment of priority pollutants, PP is not included in the current lists of regulated substances [22,23]. The absence of dedicated environmental quality standards for PP highlights the need for comprehensive ecotoxicological data to support ecological risk assessment and inform future regulatory decision-making [2,22]. An increasing body of evidence indicates that PP exerts endocrine-disrupting, reproductive, and developmental toxicities in aquatic organisms [13,24,25]. In zebrafish early developmental assays, PP was found to interfere with lipid utilization, potentially leading to neurodevelopmental and skeletal abnormalities [25]. Moreover, PP significantly altered the expression profiles of multiple mRNAs associated with neural signaling and nervous system development, including those involved in thyroid hormone transport, lipid binding, ion binding, calcium binding, endopeptidase inhibition, and hormone receptor binding [25,26]. Additional studies have reported that PP can induce feminization in *Tigriopus japonicus* [27], disrupt the antioxidant defense systems in the gills and liver of tilapia [28], and accelerate ovarian aging in adult mice [29].

*Biomphalaria glabrata* (Say, 1818), belonging to the family Planorbidae, is a common freshwater herbivorous snail. The adult shell is sinistrally coiled, with a diameter of up to approximately 40 mm and a width of about 11 mm [30]. This species is widely distributed, has limited dispersal capability, and is easy to obtain, making it widely used in laboratory studies and environmental monitoring [31]. Compared with other aquatic organisms, experiments using *B. glabrata* require less space and fewer samples, thereby reducing the cost of in vivo exposure studies [31]. Its life cycle is relatively short, taking about two months from embryo laying to the onset of reproduction in the next generation. In addition, as a hermaphroditic species, *B. glabrata* avoids the influence of sex-related response differences [32]. In developmental toxicity research, *B. glabrata* embryos offer unique advantages. This simultaneous hermaphroditic freshwater snail lays gelatinous embryo masses almost daily, each typically containing 10–40 embryos arranged in a single layer and attached to submerged substrates, allowing researchers to visually observe embryonic development throughout the entire process [31]. Embryos within a single embryo mass generally develop synchronously, allowing each embryo mass to be treated as a biological unit in toxicological assays. Under normal conditions, embryos sequentially progress through the blastula, gastrula, trochophore, veliger, and pre-hatching stage before hatching (Supplementary Figure S1A). The genome of *B. glabrata* was sequenced in 2017 [33], providing a crucial foundation for understanding its biological characteristics and immune system [34]. As an important intermediate host of *Schistosoma mansoni*, *B. glabrata* plays a key role in the transmission and geographical distribution of schistosomiasis [35]. The immune system of *B. glabrata* exhibits innate defense responses similar to those of vertebrates, enabling the recognition and elimination of various pathogens. In recent years, with increasing understanding of its immunological mechanisms, *B. glabrata* has also emerged as an important model organism in ecotoxicological research [30].

To investigate the potential effects of PP on aquatic ecosystems, this study employed *B. glabrata* as a model organism to evaluate its toxic effects across different developmental stages. By examining embryos, newly hatched snails, and adults, this study aimed to characterize stage-specific sensitivity and to assess both acute and longer-term biological responses to PP exposure. Toxic effects were evaluated with particular emphasis to survival, growth, reproduction, and tissue-level alterations to provide an integrated view of organismal responses. The findings of this study provide deeper insight into the toxic responses of this model species and an important basis for assessing the potential ecological risks of PP in freshwater ecosystems.

## 2. Materials and Methods

### 2.1. Chemicals

Propylparaben (PP, CAS No. 94-13-3), chemically named 4-hydroxybenzoic acid propyl ester, belongs to the class of parabens, with a molecular formula of C_10_H_12_O_3_ and a molecular weight of 180.20. Its melting point is 95–98 °C, boiling point approximately 297 °C, and water solubility at 20 °C is about 0.5 g/L. The PP used in this study was a crystalline solid purchased from Shanghai Macklin Biochemical Co., Ltd. (Shanghai, China), with a purity of ≥99.0%. The chemical structure of PP is shown in Supplementary Figure S1B. Its molecule contains a hydroxybenzene ring and an ester group, conferring a certain degree of lipophilicity and membrane permeability—features that may be closely related to its toxic mechanism of action. Dimethyl sulfoxide (DMSO, CAS No. 67-68-5) was obtained from Shanghai Sangon Biotech Co., Ltd. (Shanghai, China). In the experiment, 100 mg of PP was first dissolved in 1 mL of DMSO to prepare a stock solution of 100,000 mg/L, which was then serially diluted with ultrapure water to achieve the required experimental concentrations, ensuring that the final DMSO content in each treatment solution was below 0.1%. All reagents were of analytical grade and suitable for aquatic ecotoxicological experiments.

### 2.2. B. glabrata Maintenance

The *B. glabrata* used in this study were maintained in a standardized environmental control system. Specifically, transparent polystyrene containers measuring 270 × 180 × 100 mm (length × width × height) were used as rearing units, providing sufficient space for daily activities while facilitating observation and experimental management, in accordance with the quality standards for mollusk culture containers proposed by Sarkis et al. (2021) [36]. Sterilized river sand was placed at the bottom of each container to provide a suitable substrate, and sterilized polystyrene foam boards were added as oviposition substrates to simulate the natural habitat and promote embryo-laying behavior. The top of each container was covered with a transparent plastic lid to minimize water evaporation, with ventilation holes on both sides to ensure adequate air circulation.

The snails were maintained in artificial pond water (pH 7.0 ± 0.2), which had been aerated and dechlorinated and was formulated according to the artificial pond water protocol provided by the Biomedical Research Institute [37]. Under laboratory conditions, *B. glabrata* were reared at 25 ± 2 °C with a 12 h light/12 h dark photoperiod. Fresh lettuce was provided as food, with the feeding amount adjusted according to snail density and developmental stage. Culture water was replaced every 2–3 days to ensure a comfortable living environment. All snails used in the experiments were carefully selected from healthy, sexually mature individuals of uniform size, with intact shells and normal feeding and escape behaviors. Escape behavior refers to active crawling or movement away from a stimulus or contaminated area and is commonly regarded as an avoidance response to environmental stress.

### 2.3. Propylparaben Exposure

To comprehensively evaluate the toxic effects of PP on *B. glabrata*, a multi-stage experimental design was implemented, including embryonic exposure, acute toxicity assays in newly hatched and adult snails, and a long-term chronic exposure experiment combined with histopathological analysis. Ultrapure water was used as the control in all experiments. As described in Section 2.1, the final DMSO concentration in all treatments was maintained below 0.1%, which is within the range commonly used in toxicological bioassays and is generally considered unlikely to induce measurable solvent-related effects at such low concentrations [38]. Therefore, a separate solvent control was not included.

Analytical verification of PP concentrations was not conducted; therefore, all concentrations refer to nominal values. Previous studies indicate that parabens show relatively high persistence in aquatic systems [39] and limited abiotic degradation under controlled laboratory conditions [40], supporting the use of nominal concentrations in the present study.

#### 2.3.1. Acute Toxic Effects on Embryonic Developmental and Mortality of *B. glabrata*

To evaluate the acute toxicity of PP on *B. glabrata* embryos, six experimental groups were established: one control group and five PP exposure concentrations (10, 20, 30, 40, and 50 mg/L). Healthy embryo masses were collected within 12 h after oviposition by adult snails. To facilitate oviposition, sterilized polystyrene foam boards were placed in the parental culture containers as substrates. During collection, only transparent, undamaged, and morphologically intact embryo masses were selected and then randomly assigned to each treatment group. Exposure experiments were conducted in sterile 48-well plates, with 1 mL of the corresponding test solution added to each well and one embryo mass placed per well. Five replicate wells were set for each concentration, and each embryo mass was treated as a biological replicate (*n* = 5). The entire experiment was independently repeated three times under identical conditions to ensure reproducibility. All exposures were performed under controlled laboratory conditions at 25 ± 2 °C with a 12 h light/12 h dark photoperiod, and water quality was carefully maintained throughout the exposure period to minimize the influence of non-experimental factors. During the 240 h exposure, systematic observations were performed every 24 h to record the number of viable embryos, mortality, cumulative hatching rate, and developmental progression within each embryo mass. Daily observations and imaging were conducted using a Motic stereomicroscope equipped with a high-definition digital camera (Moticam S5, Motic China Group Co., Ltd., Xiamen, China), to obtain high-resolution developmental records.

Mortality during embryonic development was determined according to the following criteria: developmental arrest or delay; absence of apparent cell division or rotational movement; and morphological abnormalities, including pigment deposition, vesicle formation, and rupture of the embryo body. Differences between the treatment and control groups were compared to assess the effects of PP on embryonic development. In addition, embryonic development was continuously monitored and photographed under a stereomicroscope, and the images were digitally archived to ensure the accuracy and traceability of the data.

#### 2.3.2. Acute Toxic Effects on Newly Hatched *B. glabrata*

After completing the embryonic toxicity test, an acute exposure test was conducted using newly hatched snails to evaluate the stage-specific toxicity of PP. The concentration range was selected based on the results of the embryonic exposure test, focusing on concentrations near the observed toxicity threshold in order to better characterize acute lethality in newly hatched individuals. The embryonic exposure experiment (Section 2.3.1) was designed to assess developmental abnormalities and hatching success over a prolonged exposure period, whereas the newly hatched snail test primarily focused on short-term mortality and behavioral responses.

Healthy newly hatched *B. glabrata* were collected within 24 h after hatching. Five treatment groups were established, including four PP exposure groups at concentrations of 30, 35, 40, and 45 mg/L, and one ultrapure water control group. Each concentration consisted of three parallel replicates (*n* = 3), with 10 individuals per replicate, resulting in a total of 150 newly hatched snails. All replicates were conducted simultaneously under identical experimental conditions.

The experiment was conducted using a static exposure method in sterilized six-well cell culture plates (well capacity: 15.84 mL), with 5 mL of the corresponding test solution added to each well. This volume was selected to maintain stable exposure conditions while allowing adequate space and oxygen availability for the newly hatched snails [41]. Exposure lasted for 96 h under controlled conditions at 25 ± 2 °C with a 12 h light/12 h dark photoperiod. During the exposure period, the survival status and behavioral responses of individuals were observed and recorded every 24 h. Behavioral responses were evaluated based on locomotor activity, attachment behavior, and abnormal reactions. Mortality was determined according to the following criteria: (i) shell retraction and settling at the bottom; (ii) no response to mechanical stimulation and complete loss of movement; and (iii) marked body pallor. At the end of the exposure period, all surviving newly hatched snails were humanely euthanized. Euthanasia was performed by immersion in ice-cold water (4 °C) for at least 30 min to induce anesthesia, followed by prolonged exposure until complete cessation of movement.

#### 2.3.3. Acute and Chronic Toxic Effects on Behavioral, Survival, and Reproductive Alterations of Adult *B. glabrata*

Acute and chronic toxicity tests were conducted on adult *B. glabrata* to systematically evaluate the toxicological effects of PP. In the acute toxicity assay, five exposure concentrations (55, 56, 57, 58, and 60 mg/L) were established based on preliminary experiments to determine the 72 h LC_50_ of PP in adult snails, with ultrapure water serving as the control. All test organisms were healthy adults with shell diameters of 12–14 mm. For each concentration, five snails were maintained together in a sterile cylindrical polystyrene container (bottom diameter: 90 mm; top diameter: 116 mm) containing 300 mL of the corresponding PP solution. The experiment was independently repeated three times. During the exposure period, survival was recorded every 24 h, and systematic behavioral observations were conducted based on feeding activity, embryo masses laying, excretion, escape responses, shell retraction, and spatial distribution. Mortality was determined according to the following criteria: (i) complete immobility with no response to mechanical stimulation; (ii) hemolymph exudation or abnormal body coloration; and (iii) tissue decay or pronounced atrophy. Observations were conducted using a double-blind approach, and abnormal behavioral phenotypes were recorded photographically.

The obtained data were analyzed using nonlinear regression according to the following equation:

Y = 100/(1 + 10^[(LogLC50 − X) × HillSlope]^)

where X represents the logarithm of concentration, Y (survival rate) is the ratio of surviving individuals to the initial number, and HillSlope indicates the slope of the regression curve.

In the chronic toxicity experiment, five experimental groups were established: one control group and four PP exposure concentrations (10, 25, 45, and 49 mg/L). Each concentration group included five independent replicates (*n* = 5). Each healthy adult snail was individually maintained in a polystyrene container (volume 150 mL, top diameter 62 mm, bottom diameter 42 mm) filled with 50 mL of the corresponding test solution.

The chronic exposure period lasted for 21 days, with the test solutions renewed every three days. During the experiment, the number of embryo masses produced by *B. glabrata* was recorded daily. In addition, body weight and shell length were measured every three days using an analytical balance (precision 0.1 mg) and a digital caliper (precision 0.02 mm), respectively. Before weighing, excess water on the snail shells was gently removed using tissue. At the end of the experiment, all surviving individuals (including those in the control group) were humanely euthanized by immersion in ice-cold water (4 °C) for at least 30 min until complete loss of responsiveness, followed by confirmation of death and immediate dissection for tissue collection. Shells were carefully removed with tweezers, and only the soft tissues were retained. The soft bodies of the surviving snails from each treatment group (including controls) were placed on clean white A4 paper for macroscopic morphological observation, and their external characteristics were photographed and recorded. Subsequently, the hepatopancreas and gonadal tissues were dissected and preserved for histological sectioning to compare tissue alterations among different exposure concentrations.

### 2.4. Morphological and Histopathological Analysis of Soft Tissues in B. glabrata Exposed to Different Concentrations of PP

After the 21-day chronic exposure period, histopathological analyses of the hepatopancreas and gonads were performed. Tissues from the hepatopancreas and gonads were randomly selected from the five treatment groups (one control group and four PP exposure concentrations (10, 25, 45, and 49 mg/L)) for histological preparation. Histological processing, including fixation, paraffin embedding, sectioning, and hematoxylin–eosin (HE) staining, was carried out by a professional histological service provider.

Samples were fixed in 4% neutral buffered formalin (NBF) for 24 h, followed by graded ethanol dehydration, xylene clearing, and paraffin embedding. After embedding, the tissue blocks were sectioned into continuous slices with a thickness of 3 μm. The HE staining procedure was conducted as follows: tissue sections were dewaxed in xylene I and II for 20 min each, then rehydrated through a graded ethanol series—absolute ethanol (twice, 5 min each), 95% ethanol (5 min), 85% ethanol (5 min), and 75% ethanol (5 min)—followed by rinsing with tap water. Staining was performed with hematoxylin for 3–5 min, followed by differentiation and bluing, and then counterstaining with eosin (in 95% ethanol) for 15–30 s. The sections were subsequently dehydrated in absolute ethanol I, II, and III (2 min each), cleared in n-butanol I, II, and xylene I, II (2 min each), and finally mounted with neutral resin. After staining, the slides were scanned at high resolution (1–800×) using a bright-field digital slide scanner at a commercial histological service facility. Observations and image examination were performed using SlideViewer software provided by Wuhan Borefu Biotechnology Co., Ltd. (Wuhan, China).

### 2.5. Statistical Analysis

All statistical analyses were performed using GraphPad Prism software (version 10). The LC_50_ values in the acute and embryonic toxicity tests were calculated by nonlinear regression (curve fitting) using the model “log(inhibitor) vs. normalized response—variable slope,” with the confidence interval set at 95% [42]. In the chronic toxicity tests, data on growth rate (weight and shell length increase) and embryo mass production were analyzed using two-way analysis of variance (Two-way ANOVA) to evaluate the interactive effects of concentration and exposure time. When necessary, Tukey’s multiple comparison test was applied to compare differences among groups. In addition, independent-sample *t*-tests were performed to assess differences between the control and treatment groups.

Before performing parametric tests, the Shapiro–Wilk test and Levene’s test were applied to verify data normality and homogeneity of variances, respectively [43]. If the data did not meet the assumptions of parametric tests, corresponding nonparametric methods were used. Experimental data were considered valid only when the mortality rate of the control group was below 10% [44]. Unless otherwise specified, all results are presented as mean ± standard deviation (mean ± SD). Significant differences between groups were indicated by asterisks in the figures: * *p* < 0.05, ** *p* < 0.01, *** *p* < 0.001, **** *p* < 0.0001. All statistical analyses were two-tailed, with the significance level set at α = 0.05, and *p* < 0.05 was considered statistically significant.

The overall experimental design, including experiment, exposure type, duration and concentrations, is summarized in Table 1.

## 3. Results

### 3.1. Acute Toxic Effects of PP on the Embryonic Development of B. glabrata

**Figure 1:**
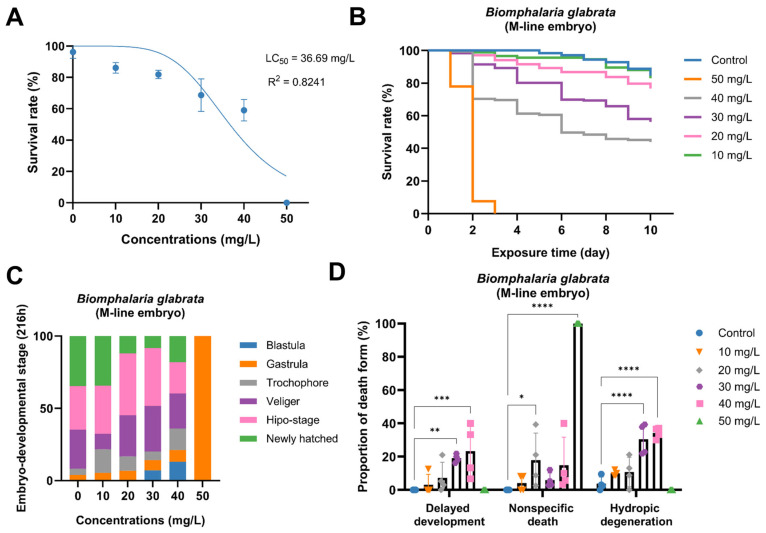
Acute toxic effects of PP on *Biomphalaria glabrata* embryos. (**A**) Nonlinear regression analysis of embryo survival rate versus PP concentration after 168 h exposure. Blue dots represent the observed data points, and the blue line represents the fitted nonlinear regression curve, indicating the LC_50_ value and R^2^. (**B**) Survival curves of embryos exposed to different PP concentrations over a 10-day period. (**C**) Distribution of embryonic developmental stages after 216 h exposure to different PP concentrations. (**D**) Proportions of different types of embryonic death after 168 h exposure under various PP concentrations. Significance levels are indicated by asterisks: * *p* < 0.05, ** *p* < 0.01, *** *p* < 0.001, **** *p* < 0.0001.

A 240 h continuous exposure experiment was conducted to evaluate the developmental toxicity of PP during the embryonic stage. Based on survival data at 168 h yielded an LC_50_ value of 36.69 mg/L (R^2^ = 0.8241). As PP concentration increased, embryonic survival rate showed a clear downward trend: at 10 mg/L, there was no significant difference compared with the control group; in the range of 20–40 mg/L, survival rate decreased progressively with increasing concentration; and at 50 mg/L, embryo survival dropped to 0%. These results indicate that PP exhibits a clear dose-dependent toxicity during the embryonic stage (Figure 1A). Temporal survival analysis further demonstrated that the toxic effects of PP intensified with prolonged exposure time. In particular, the 50 mg/L group showed rapid mortality, with survival dropping to 0% by day 3, while lower concentrations exhibited more gradual declines (Figure 1B). Developmental stage distribution analysis revealed a graded inhibition of embryogenesis. At 10 mg/L, most embryos were able to complete development, with only a few arrested at the trochophore stage. At 20 mg/L, marked developmental delay occurred, with most embryos arrested at the blastula and veliger stages. At 30 mg/L, arrest at the blastula and gastrula stages increased substantially. At 40 mg/L, nearly all embryos were blocked at early developmental stages, and progression to later stages was rarely observed. At 50 mg/L, all embryos were arrested at the gastrula stage and failed to develop further (Figure 1C). Statistical analysis of embryo mortality types further characterized the toxic manifestations. Three types of mortality—developmental delay, nonspecific death (death of embryos without clear morphological abnormalities or developmental arrest), and hydropic degeneration—all increased in frequency with rising concentration. Developmental delay was significantly elevated in the 20–40 mg/L groups; nonspecific death peaked at 30 mg/L, slightly decreased at 40 mg/L, and rose again to nearly 100% at 50 mg/L; hydropic degeneration mainly occurred at ≥30 mg/L and generally increased with concentration. In contrast, abnormalities were rarely observed in the control group (Figure 1D).

**Figure 2:**
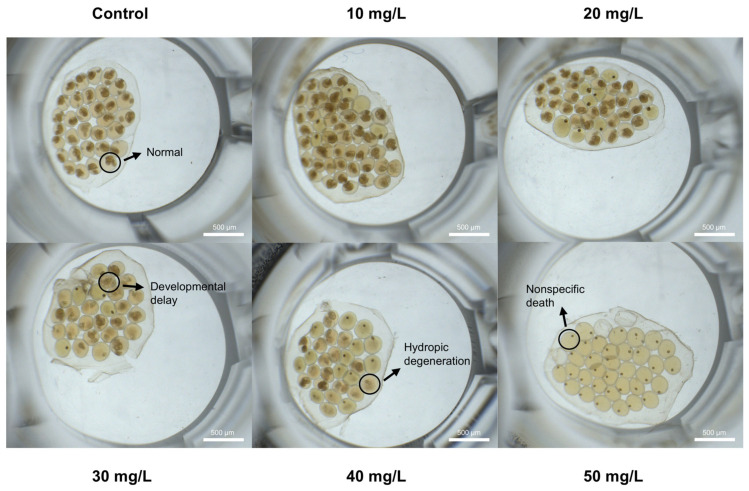
Representative morphological alterations of *Biomphalaria glabrata* embryos following acute exposure to PP. Embryo masses were exposed to one control group and different PP concentrations (10, 20, 30, 40, and 50 mg/L) for 120 h. Representative images illustrate normal development in the control group and typical morphological changes observed at increasing PP concentrations (indicated by arrows and circles). Images were captured under identical conditions, scale bar = 500 µm.

Microscopic images are shown in Figure 2. Embryos in the control group developed normally with well-defined structures. Under 10 mg/L PP exposure, most embryos were similar to the control, showing only slight developmental delay. At 20–30 mg/L, some embryos exhibited developmental arrest and pigmentation abnormalities. At 40 mg/L, developmental inhibition became more pronounced, with cellular structures appearing increasingly blurred. At 50 mg/L, all embryos displayed pronounced developmental arrest and complete loss of normal morphology. These images clearly illustrate the concentration-dependent toxic effects of PP on the embryonic development of *B. glabrata*.

### 3.2. Acute Toxic Effects of PP on Newly Hatched B. glabrata

**Figure 3:**
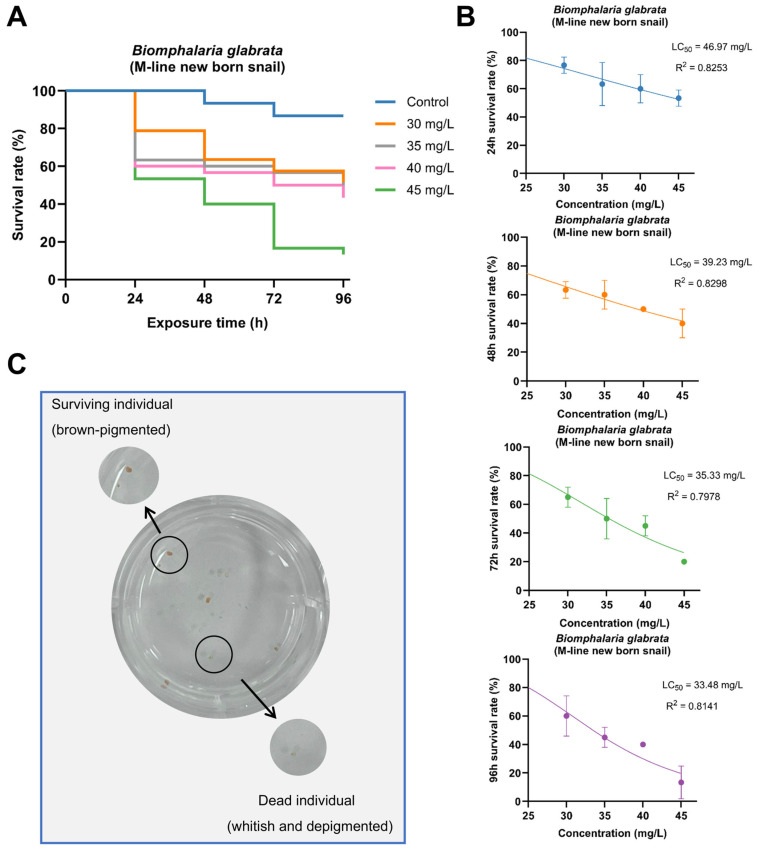
Acute toxic effects of PP on newly hatched *Biomphalaria glabrata*. (**A**) Survival of *B. glabrata* newly hatched snails exposed to different PP concentrations over a 96 h period; (**B**) From top to bottom, nonlinear regression analyses of concentration-survival relationships for newly hatched snails exposed to PP for 24 h, 48 h, 72 h, and 96 h, respectively, showing LC_50_ and R^2^ values; (**C**) Morphological differences between surviving individuals (brown, top left, indicated by arrows and black circles) and dead individuals (depigmented, bottom, indicated by arrow and black circle).

In the acute exposure experiment with newly hatched *B. glabrata*, PP exhibited a clear dose-dependent toxic effect. Survival curves showed that survival rates progressively decreased with increasing concentration and exposure duration. In the 30–35 mg/L groups, survival remained relatively high within the first 48 h but declined markedly at 72–96 h; in contrast, individuals in the 40–45 mg/L groups showed a much faster mortality trend, with survival rates at 96 h significantly lower than those in the lower-concentration and control groups (Figure 3A). The LC_50_ values calculated from the concentration-response relationship for 24 h, 48 h, 72 h, and 96 h were 46.97 mg/L, 39.23 mg/L, 35.33 mg/L, and 33.48 mg/L, respectively, with R^2^ values of 0.8253, 0.8298, 0.7978, and 0.8141 (Figure 3B). Individuals in the control group maintained normal locomotor activity and attachment ability throughout the experiment, whereas those in higher-concentration groups showed progressively reduced mobility and a rapid decline in number over time.

In addition, regarding morphological characteristics (Figure 3C), surviving individuals typically retained brown pigmentation and were able to attach to the bottom or edges of the culture wells. In contrast, dead individuals exhibited pale coloration, complete pigment loss, and settled at the bottom, showing no response to external stimuli. This distinct morphological difference provided a clear visual basis for distinguishing between live and dead individuals.

### 3.3. Acute Toxic Effects of PP to Adult B. glabrata

**Figure 4:**
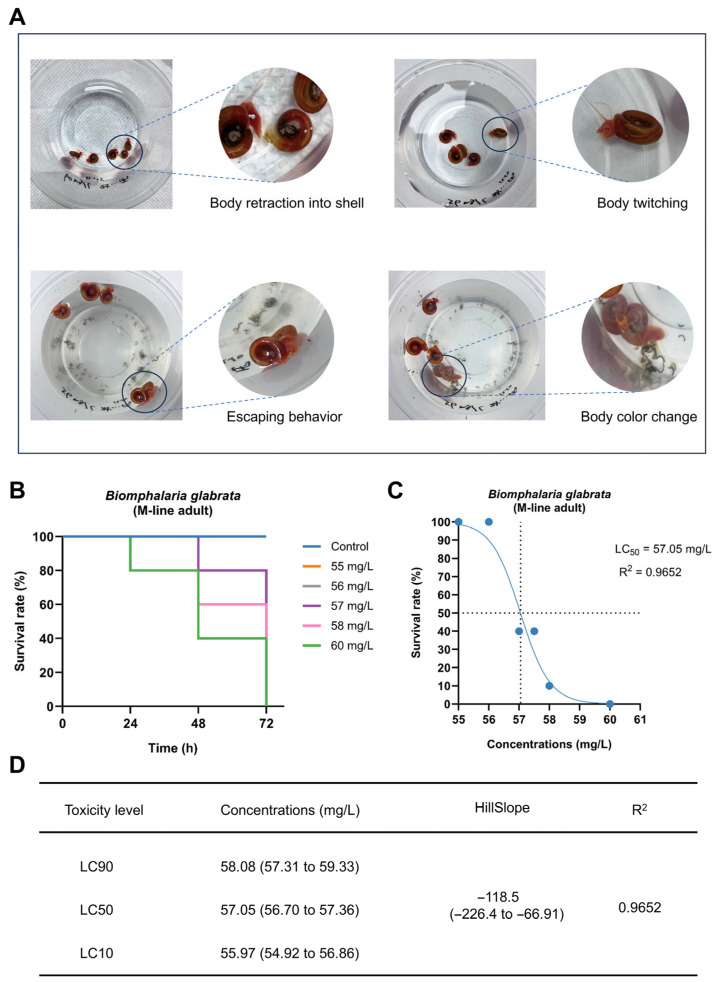
Acute toxic effects of PP on adult *Biomphalaria glabrata*. (**A**) Representative behavioral and morphological responses of adult *B. glabrata* under high concentrations of PP exposure; circles indicate regions magnified in adjacent panels, with dashed lines connecting original and magnified views. (**B**) Survival curves of adult *B. glabrata* during 72 h exposure to different PP concentrations. The curves of the 55 and 56 mg/L groups overlap completely with that of the control group and are therefore not visually distinguishable; (**C**) Nonlinear regression analysis of dose-survival rate for adult *B. glabrata* after 72 h PP exposure. Data points represent observed survival percentages. The LC_50_ value is indicated by dashed lines, and the coefficient of determination (R^2^) is shown; (**D**) LC_10_, LC_50_, and LC_90_ values derived from the dose-survival regression curve, with corresponding 95% confidence intervals, HillSlope, and R^2^.

Under high PP exposure concentrations, adult snails exhibited a series of obvious behavioral and physiological abnormalities, including body retraction into the shell, body twitching, frequent escaping behavior, gradual lightening of body color from reddish-brown, and increased mucus secretion (Figure 4A).

In the 72 h exposure experiment, the survival rate of *B. glabrata* showed a clear concentration-dependent decline across treatment groups. Kaplan–Meier survival curves indicated a significant increase in mortality at concentration was ≥56 mg/L (Figure 4B). Nonlinear regression analysis based on the dose–response relationship revealed that the 72 h LC_50_ for adult snails was 57.05 mg/L, with a 95% confidence interval of 56.70–57.36 mg/L, showing a high goodness of fit (R^2^ = 0.9652) (Figure 4C,D). In addition, the calculated LC_10_ and LC_90_ values were 55.97 mg/L (95% confidence interval [CI]: 54.92–56.86) and 58.08 mg/L (95% CI: 57.31–59.33), respectively (Figure 4D).

### 3.4. Chronic Toxic Effects of PP on Growth and Hepatopancreatic Tissue in B. glabrata

**Figure 5:**
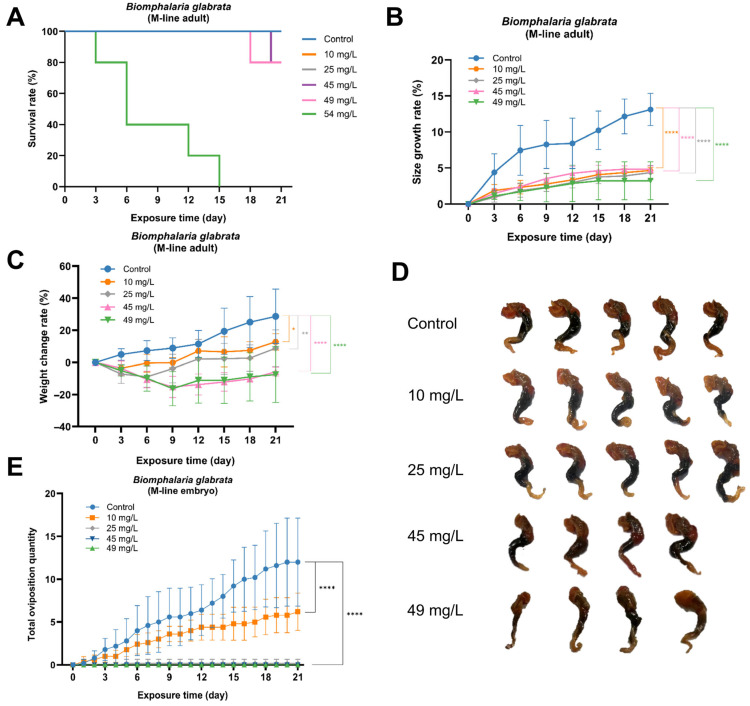
Effects of chronic PP exposure on the growth, morphology, and histology of adult *Biomphalaria glabrata*. (**A**) Survival curves of *B. glabrata* under different PP concentrations during the 21-day exposure period. The curves of the 10 mg/L and 25 mg/L groups overlap completely with that of the control group and are therefore not visually distinguishable in the figure. (**B**) Shell length growth rate of adult *B. glabrata* under different PP concentrations during exposure. (**C**) Body weight change rate of adult *B. glabrata* under different PP concentrations during exposure. (**D**) Representative external morphology of adult *B. glabrata* at the end of exposure under different PP concentrations. (**E**) Cumulative number of embryo masses produced by adult *B. glabrata* under different PP concentrations during the exposure period. Asterisks indicate significant differences compared with the control group at the same time point (* *p* < 0.05, ** *p* < 0.01, **** *p* < 0.0001).

During the 21-day chronic exposure experiment, the survival rate of *B. glabrata* exhibited a pronounced concentration-dependent pattern. At the highest exposure concentration (54 mg/L), all individuals died before day 15. Due to complete mortality before day 15, individuals in the 54 mg/L group were not included in subsequent growth and reproduction assessments. Partial mortality occurred in the medium-concentration groups (49 mg/L and 45 mg/L), whereas all snails in the low-concentration groups (25 mg/L and 10 mg/L) survived until the end of the experiment (Figure 5A). Growth indicators were also markedly inhibited. In terms of shell length, individuals in the 49 mg/L and 45 mg/L groups showed almost no further growth, while those in the 25 mg/L and 10 mg/L groups exhibited limited increases but remained significantly lower than those in the control group. Regarding body weight, individuals in the medium- and high- concentration groups displayed a sharp decrease during the early phase (0–6 days), followed by a slight rebound that, however, did not return to the initial level. In contrast, the low-concentration groups showed a mild initial decline, then gradually recovered, ultimately exceeding their baseline weight by the end of the exposure period (Figure 5B,C).

**Figure 6:**
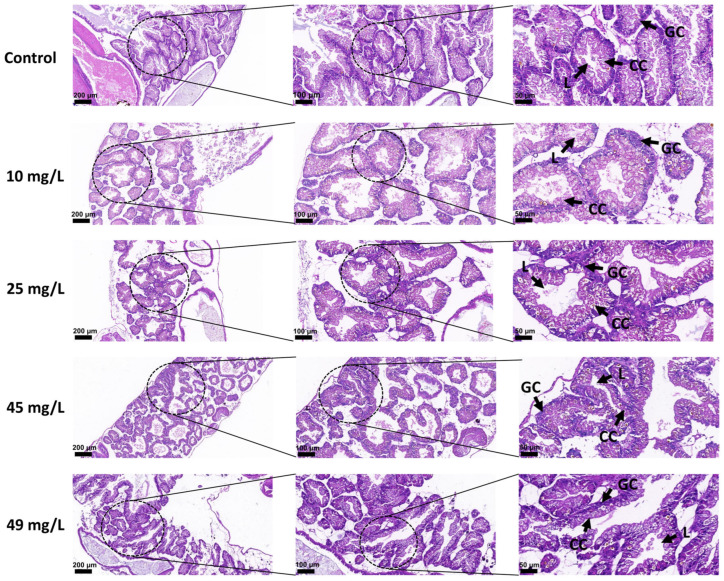
Histopathological alterations in the hepatopancreas of adult *Biomphalaria glabrata* exposed to one control group and different concentrations of PP. Representative HE-stained sections of the hepatopancreas after 21 days of exposure (from top to bottom: control, 10, 25, 45, and 49 mg/L). Dotted circles indicate the magnified regions shown in the adjacent right panels. Scale bars are shown in each panel. Arrows indicate representative structures. Abbreviations: L, lumen; GC, granular cells; CC, columnar cells.

For morphological observation, the shells of all *B. glabrata* individuals were removed. Compared with the control group, no severe external deformities were observed in the treated snails; however, individuals in the medium- and high-concentration groups exhibited slight darkening of body coloration and reduced tissue integrity (Figure 5D). Histological results revealed that PP exposure induced pathological alterations in the hepatopancreatic tissue. In the control group, acinar structures were well organized with clear boundaries, homogeneous cytoplasm in digestive cells, distinct nuclei, and intact lumina. At 10 mg/L, slight alterations were observed, including blurred acinar boundaries and mild cytoplasmic heterogeneity in some digestive cells, although the overall structure remained relatively intact. At 25 mg/L, the acinar disorganization became more pronounced, and lumina were notably dilated. In the 45 mg/L group, acinar structures were disordered, luminal dilation increased, and acinar boundaries became indistinct. Most digestive cells exhibited vacuolar degeneration and cytoplasmic loosening, resulting in an overall relaxed tissue structure, indicating functional impairment of the hepatopancreas. The most severe damage was observed at 49 mg/L, where acinar structures were extensively disrupted, boundaries were lost, and cell polarity disappeared. Numerous digestive cells displayed severe vacuolar degeneration and necrosis, while secretory cells showed cytoplasmic disorganization and incomplete luminal structures, accompanied by widened intercellular spaces, suggesting damage to the extracellular matrix (Figure 6).

### 3.5. Chronic Toxic Effects of PP on Reproduction and Gonad Histopathology in B. glabrata

During the 21-day observation period, the reproductive activity of *B. glabrata* was significantly inhibited by PP exposure. The control group maintained normal reproductive activity, with embryo mass deposition occurring almost daily. In contrast, only the 10 mg/L exposure group exhibited limited reproductive behavior, and the number of embryos masses produced was markedly lower than that in the control group. No embryo mass deposition was observed in the 25, 45, or 49 mg/L exposure groups (Figure 5E).

**Figure 7:**
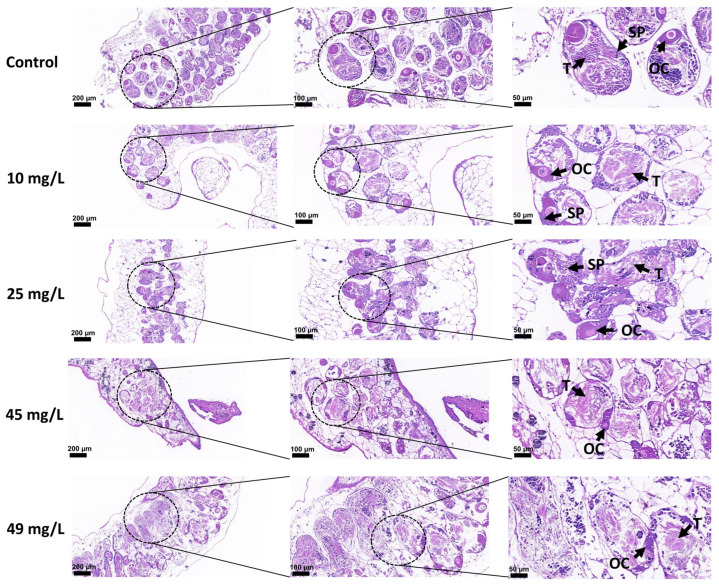
Histopathological effects of PP exposure on the gonads of adult *Biomphalaria glabrata*. Representative HE-stained sections of gonadal tissues after 21 days of exposure (from top to bottom: control, 10, 25, 45, and 49 mg/L). Dotted circles indicate the magnified regions shown in the adjacent right panels. Scale bars are shown in each panel. Arrows indicate representative structures. Abbreviations: OC, oocytes; SP, spermatozoa; T, testis.

To investigate the histological basis of the observed reproductive inhibition, HE staining was performed on the gonadal tissues of *B. glabrata*. The results revealed concentration-dependent structural damage to the gonads following PP exposure. In the control group, gonadal structures were intact: oocytes in the ovary were densely arranged and morphologically regular, with homogeneous cytoplasm and evenly distributed chromatin; the testis exhibited clearly defined seminiferous tubules containing a normal number of spermatogonia and well-developed spermatozoa, indicating normal reproductive function. In the 10 mg/L exposure group, slight alterations were observed: some oocytes showed cytoplasmic irregularities, and the number of spermatozoa showed a minor decline, though the overall structure remained largely intact. In the 25 mg/L group, the number of oocytes decreased and some displayed abnormal morphology; in the testis, spermatogonia were sparsely distributed and spermatogenesis efficiency declined. In the 45 mg/L group, damage became more pronounced, with evident oocyte shrinkage and degeneration, cytoplasmic vacuolation, and a marked reduction in spermatogonia, accompanied by arrested sperm development. In the 49 mg/L exposure group, gonadal damage was most severe: massive necrosis and vacuolar degeneration occurred in oocytes, chromatin distribution was disrupted, some follicular cavities collapsed, and testicular boundaries became indistinct, with extensive germ cell necrosis and detachment, resulting in nearly complete inhibition of sperm formation (Figure 7).

## 4. Discussion

The present study demonstrates that PP induces significant toxic effects in *B. glabrata* across multiple life stages, including reduced survival, inhibited growth, hepatopancreatic damage, and, most notably, severe reproductive impairment. Among these effects, the pronounced inhibition of reproduction and the distinct histopathological alterations in gonadal tissues are particularly noteworthy, as they suggest potential population-level consequences of chronic PP exposure. These results provide new evidence that PP not only affects survival and growth but may also disrupt reproductive capacity in freshwater gastropods, highlighting the ecological relevance of long-term exposure. By integrating evidence from life-history traits, histopathology, and reproductive endpoints, this study underscores reproductive toxicity as a key component of PP toxicity in freshwater gastropods.

Parabens are a group of structurally similar preservatives whose toxicity and antimicrobial activity generally increase with the length of the alkyl chain [3,45]. Previous studies have reported distinct toxicity levels among different paraben monomers in various aquatic organisms. In zebrafish embryo assays, the LC_50_ values of MP, EP, and PP based on embryo mortality and malformation rates at 120 h post-fertilization (hpf) were 71.25, 32.6, and 11.1 mg/L, respectively, indicating that EP and PP are more toxic than MP, with PP exhibiting the strongest toxicity [26]. In the marine copepod *T. japonicus*, both EP and PP significantly reduced growth rates and altered sex ratios, whereas MP had relatively weaker effects [27]. Biochemical studies in freshwater fish have shown that exposure to MP, EP, and PP can disrupt antioxidant-related enzymes, such as superoxide dismutase (SOD), glutathione peroxidase (GPx), and glutathione reductase (GR) in *Oreochromis niloticus*, though not always accompanied by marked lipid peroxidation [28]. Similarly, experiments with *Oncorhynchus mykiss* revealed that MP induces dose-dependent hepatic and renal tissue damage, increased malondialdehyde (MDA) levels, and decreased GPx activity [46]. Furthermore, in acute toxicity tests with the freshwater crustacean *Daphnia magna*, the 24 h EC_50_ of MP was approximately 45 mg/L, whereas that of PP was about 11 mg/L, again demonstrating that PP exhibits markedly higher toxicity than MP [20].

The results of our embryonic developmental toxicity test showed that the 168 h LC_50_ for embryos was approximately 36.69 mg/L, whereas the acute toxicity tests revealed that the 96 h LC_50_ for newly hatched snails and the 72 h LC_50_ for adults were approximately 33.48 mg/L and 57.05 mg/L, respectively, demonstrating that PP induces significant toxic effects at multiple life stages of *B. glabrata*. Regarding chronic toxicity, even at sublethal concentrations, PP markedly inhibited growth and weight recovery. In the medium-high concentration groups (45–49 mg/L), body weight decreased significantly during the early phase and recovered only slightly later, while shell growth nearly stagnated. Although the low concentration groups (10–25 mg/L) showed slight recovery, their growth remained significantly lower than that of the control. This finding in our study is consistent with observations in other aquatic organisms: prolonged exposure to high concentrations of toxicants often leads to chronic stress, resulting in suppressed feeding activity and disrupted energy metabolism, which manifest as reduced or even negative growth. For instance, one study reported that zebrafish exposed to low concentrations (0–25 μM) of butylated hydroxyanisole showed no significant change in body length, whereas exposure to higher concentrations (50 and 100 μM) led to a significant reduction in body length compared with the control group [47]. Another study demonstrated that chronic exposure to tributyltin caused reduced energy reserves and deteriorated health status in the *Ostrea edulis*: after 9 weeks of exposure to 2.0 µg/L tributyltin, the condition index decreased significantly (*p* < 0.05), while no significant changes were observed at 0.02 µg/L or 0.2 µg/L [48]. Based on these findings, it can be inferred that exposure to higher concentrations of PP places *B. glabrata* under prolonged stress, leading to pronounced physiological responses and deteriorated health, which ultimately result in minimal shell growth and reduced body weight.

The histological analysis in the present study further revealed a clear relationship between hepatopancreatic damage and chronic toxicity. We found that as the concentration of PP increased, pathological alterations in the hepatopancreas became progressively severe, ultimately leading to structural disorganization and a significant impairment of physiological function. Previous studies have shown that PP exposure can cause a marked elevation in both plasma vitellogenin levels and its synthesis rate in the liver of *O. mykiss* [49]. As the central organ responsible for detoxification, metabolism, and immune regulation, the liver is often the primary target of chemical toxicity [50]. In mollusks, the hepatopancreas serves an analogous function, making it a critical target organ for chronic chemical exposure. Literature evidence has also confirmed the hepatotoxic nature of PP, with its mechanisms involving disturbances in energy metabolism, increased superoxide anion production, and the induction of apoptosis [51]. For example, exposure to 4.0 mg/L PP has been shown to trigger oxidative stress in hepatocytes of *Oreochromis niloticus*, manifested by a decrease in total glutathione levels after 6 days of exposure followed by an increase after 12 days [51]. Moreover, during the embryonic development of *Echeneis naucrates*, exposure to 4.0 mg/L PP resulted in liver atrophy and neuronal degeneration [13]. In *Gambusia affinis*, histopathological alterations in the liver were observed after only 4 days of exposure to 0.15–240 μg/L PP, and severe damage was evident after 32 days, including hepatic sinus dilation, cytoplasmic vacuolation, cell lysis, and nuclear aggregation [52].

In addition to its inhibitory effects on growth and development, the reproductive impairment observed in this study was the most striking and ecologically relevant finding. During the 21-day exposure period, the control group maintained stable embryo mass- laying activity, whereas only a few embryo masses were produced in the 10 mg/L group; no embryo mass production was observed in the 25, 45, or 49 mg/L groups. Histological sections of the gonads revealed clear dose-dependent pathological changes: cytoplasmic heterogeneity, reduced numbers of oocytes, and sparse spermatogonia at 10–25 mg/L; oocyte shrinkage/degeneration and arrested spermatogenesis at 45 mg/L; and seminiferous tubule boundary disruption, extensive oocytes vacuolation, and tissue disorganization at 49 mg/L. PP has been shown to exert reproductive toxicity across a variety of animal models, consistent with our observations. Similar effects have been observed in invertebrates, including *Caenorhabditis elegans* [53], *Daphnia magna* [24], *Drosophila melanogaster* [54]. These chronic toxic effects are likely closely related to oxidative stress, as previous studies have confirmed that PP can induce the generation of reactive oxygen species (ROS), thereby impairing antioxidant defense mechanisms [55,56]. Endocrine disruption has also been recognized as a major mechanism underlying PP toxicity, contributing not only to reproductive defects but also to imbalances in sex ratio and developmental delays [57]. Similar findings have been reported in fish species: for example, *Clarias batrachus* exposed to malathion exhibited growth retardation, abnormal metabolic hormone levels, and altered body weight [58], while *Danio rerio* exposed to endosulfan showed reduced reproductive capacity, slower growth, and delayed sexual maturation [59]. Moreover, the chemical structure of PP resemblance that of estrogen, which may enhance its endocrine-disrupting effects, particularly in males [60,61]. Previous studies have found that PP can delay the reproductive cycle of the marine copepod *T. japonicus* and induce feminization [27]. Taken together, we infer that the pronounced inhibition of reproductive capacity in *B. glabrata* exposed to PP is likely associated with oxidative stress and endocrine disruption, as suggested by previous studies.

Overall, the results indicate that chronic PP exposure can affect key physiological and reproductive functions in freshwater gastropods, suggesting that sublethal effects may contribute to long-term ecological consequences that are not reflected by acute mortality alone. These findings highlight the importance of considering reproductive endpoints and tissue-level alterations when evaluating the ecological risks of paraben contamination. Further studies under environmentally relevant exposure conditions would help to better understand the long-term impacts of PP in natural freshwater systems.

## 5. Conclusions

This study systematically evaluated the toxicity of PP in the freshwater snail *B*. *glabrata* at different developmental stages under both acute and chronic exposure conditions. Acute exposure resulted in clear concentration-dependent mortality and developmental impairment, with embryos and newly hatched snails showing higher sensitivity than adults, indicating life-stage differences in susceptibility. Chronic exposure to sublethal concentrations significantly inhibited growth and reproduction in adult snails, and histopathological analyses revealed progressive structural damage in the hepatopancreas and gonadal tissues. These morphological alterations provide anatomical evidence supporting the observed functional impairments. Behavioral responses, including shell retraction, body twitching, and escape behavior, were observed under high concentrations of PP and are considered typical stress responses of gastropods to chemical exposure.

Taken together, the results demonstrate that PP can affect survival, development, reproduction, behavior, and tissue integrity in *B. glabrata*, and that sublethal concentrations may produce biologically significant effects that are not detectable through short-term mortality endpoints alone. Such effects may have implications for population maintenance and ecosystem stability in freshwater environments. Future studies should focus on elucidating the molecular and physiological mechanisms underlying PP toxicity and evaluating ecological risks under environmentally relevant exposure scenarios.

## Figures and Tables

**Table 1:** Summary of acute and chronic toxicity experiments conducted on *Biomphalaria glabrata*.

Experiment	Exposure Type	Duration	Experimental Groups
Embryo test	Acute	240 h	Control and PP concentrations of 10, 20, 30, 40, 50 mg/L
Newly hatched snail test	Acute	96 h	Control and PP concentrations of 30, 35, 40, 45 mg/L
Adult acute	Acute	72 h	Control and PP concentrations of 55, 56, 57, 58, 60 mg/L
Adult chronic	Chronic	21 days	Control and PP concentrations of 10, 25, 45, 49 mg/L
Histology	Chronic	21 days	Control and PP concentrations of 10, 25, 45, 49 mg/L

## Data Availability

The original contributions presented in this study are included in the article/Supplementary Material. Further inquiries can be directed to the corresponding author.

## Supplementary Materials

The following supporting information can be downloaded at: https://www.mdpi.com/article/10.3390/toxics14030203/s1, Figure S1: Embryonic development stages of *Biomphalaria glabrata* and chemical structure of propylparaben. (A) Distribution of embryonic developmental stages at 216 h under different PP concentrations. (B) The left panel shows the chemical structure of PP, while the right panel presents its model.
