# Supplementary material for: Acute and Chronic Toxicity of Propylparaben in the Freshwater Snail Biomphalaria glabrata: Effects on Survival, Growth, Reproduction, and Histopathology
Source: Toxics. 2026 Feb 27;14(3):203. doi: 10.3390/toxics14030203 (PMC13030631; doi:10.3390/toxics14030203)
Supplement: Supplementary file 1 [file toxics-14-00203-s001.zip › toxics-4151354-supplementary.pdf]

Article

Acute and Chronic Toxicity of Propylparaben in the Freshwater Snail *Biomphalaria glabrata*: Effects on Survival, Growth, Reproduction, and Histopathology

Qingzhi Zhao [1,†], Yutong Zhao [1,†], Jiyuan Wang [1,2], Jialu Xu [1], Hairun Li [1], Xinyi Fei [1], Yijie Zhang [1], Ruke Wang [1], Yuqing Shao [1], Anni Jin [1], Hao Wu [1], Lailing Du [1], Xiaofen Zhang [1], Huiliang Zou [3], Hongyu Li [1,*] and Xiaoling Xu [1,*]

1 Key Laboratory of Artificial Organs and Computational Medicine in Zhejiang Province, Shulan International Medical College, Zhejiang Shuren University, Hangzhou 310015, China; qingzhizhao33@gmail.com (Q.Z.); yutong_zhao2022@126.com (Y.Z.); wangjiyuan@hmc.edu.cn (J.W.); xujialuuuuu@gmail.com (J.X.); lihairun0521@gmail.com (H.L.); 18506730316@139.com (X.F.); zhangyijie1101@gmail.com (Y.Z.); wangruke111@gmail.com (R.W.); yuqingshao2@gmail.com (Y.S.); annijin4719@gmail.com (A.J.); wwu28789@gmail.com (H.W.); dulailing@zjsru.edu.cn (L.D.); jxxfzhang@126.com (X.Z.)

2 School of Basic Medicine and Forensic Medicine, Hangzhou Medical College, Hangzhou 310053, China

3 Huzhou Institute for Food and Drug Control, Huzhou 313000, China; zouhuiliang@163.com

* Correspondence: hongyu88926@zjsru.edu.cn (H.L.); ziyao1988@zju.edu.cn (X.X.)

† These authors contributed equally to this work.

Supplementary material

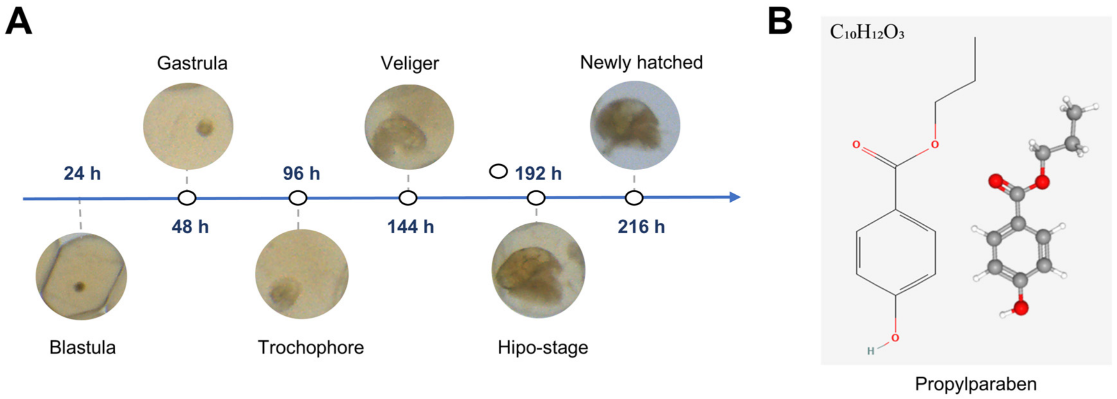

Figure S1: Embryonic development stages of *Biomphalaria glabrata* and chemical structure of propylparaben. (A) Distribution of embryonic developmental stages at 216 h under different PP concentrations. (B) The left panel shows the chemical structure of PP, while the right panel presents its model.
